# Measuring the impact of suspending Umrah, a global mass gathering in Saudi Arabia on the COVID-19 pandemic

**DOI:** 10.1007/s10588-021-09343-y

**Published:** 2021-09-06

**Authors:** Sultanah M. Alshammari, Waleed K. Almutiry, Harsha Gwalani, Saeed M. Algarni, Kawther Saeedi

**Affiliations:** 1 Department of Computer Science, King Abdulaziz University, Jeddah, Saudi Arabia; 2 Department of Mathematics, College of Arts and Science in Ar Rass, Qassim University, Qassim, Saudi Arabia; 3 Department of Computer Science and Engineering, University of North Texas, Denton, TX USA; 4 Saudi Center for Disease Prevention and Control, Jeddah, Saudi Arabia; 5 Department of Information Systems, King Abdulaziz University, Jeddah, Saudi Arabia

**Keywords:** Infectious diseases, Coronavirus, COVID-19, Mass gatherings, Umrah, Hajj, Travel restriction, Epidemic modeling, SEIR model

## Abstract

Since the early days of the coronavirus (COVID-19) outbreak in Wuhan, China, Saudi Arabia started to implement several preventative measures starting with the imposition of travel restrictions to and from China. Due to the rapid spread of COVID-19, and with the first confirmed case in Saudi Arabia in March 2019, more strict measures, such as international travel restriction, and suspension or cancellation of major events, social gatherings, prayers at mosques, and sports competitions, were employed. These non-pharmaceutical interventions aim to reduce the extent of the epidemic due to the implications of international travel and mass gatherings on the increase in the number of new cases locally and globally. Since this ongoing outbreak is the first of its kind in the modern world, the impact of suspending mass gatherings on the outbreak is unknown and difficult to measure. We use a stratified SEIR epidemic model to evaluate the impact of Umrah, a global Muslim pilgrimage to Mecca, on the spread of the COVID-19 pandemic during the month of Ramadan, the peak of the Umrah season. The analyses shown in the paper provide insights into the effects of global mass gatherings such as Hajj and Umrah on the progression of the COVID-19 pandemic locally and globally.

## Introduction

A novel Coronavirus disease, COVID-19, emerged in Wuhan, China on December 2019. By early 2020, the COVID-19 infections were exported to various countries outside China, including Singapore, Japan, South Korea, Australia, and Germany via international travel (Singhal 2020). Since then, many countries enforced travel restrictions to and from China to prevent the spread of the virus across the world (Sohrabi et al. 2020). However, by March 11, 2020, the World Health Organization (WHO) declared the COVID-19 outbreak as a pandemic (World Health Organization 2020b). In the early stages of the pandemic, affected countries responded to the increasing number of COVID-19 confirmed cases by implementing several preventive measures to control the spread of the disease. These measures have included travel restrictions, isolation of infected populations, quarantine of suspected cases, cancellation of major events, school closure, and public lockdown (Anderson et al. 2020; Bedford et al. 2020; McCloskey et al. 2020).

While the first travel-related case of COVID-19 in Saudi Arabia was reported on the 2nd of March 2020, traveling to or from China was restricted by the Saudi government on February 6, 2020. Further travel restrictions were issued against several affected countries including Italy, France, Germany, Turkey, and Spain as the COVID-19 outbreak extended outside China affecting several regions across the world. Due to the extensive spread of the disease worldwide, Saudi Arabia decided to suspend one of the major global gatherings in Saudi Arabia, the Umrah pilgrimage to Mecca (also spelled Makkah), for both international and domestic pilgrims on February 27, 2020.

In addition to local and regional sport events, and other gatherings, Saudi Arabia hosts two major global mass gatherings, Hajj and Umrah. Both religious pilgrimages associated with the arrival of millions of Muslims from all over the world. According to GASTAT, the Saudi General Authority for Statistics (General Authority for Statistics 2020), almost 2.5 million pilgrims completed Hajj with international participants making up for more than 70% of the attendance, and about 19 million pilgrims performed Umrah throughout the year with 7.5 million pilgrims arriving from outside of Saudi Arabia in 2019. Such mass gatherings can pose a significant threat to controlling the extent of the epidemic in Saudi Arabia and in the home countries of the returning participants (Atique and Itumalla 2020; Ebrahim and Memish 2020). Due to the increasing health risks associated with MGs, the WHO is working directly with host countries and organizers of these events for effective preparedness and response planning during the COVID-19 pandemic (World Health Organization 2020c). Canceling or postponing global mass gatherings remains to be the recommended countermeasure to contain the COVID-19 pandemic due to the absence of an approved vaccine for the disease (Ahmed and Memish 2020; Ebrahim and Memish 2020). However, canceling or postponing international pre-planned mass gatherings, such as Olympics games or World Cup events may lead to huge economic losses while negatively affecting the livelihoods of people whose incomes are directly dependent on these events. Host nations spend billions of dollars of money for planning and preparing for these events, and a decision of cancellation or postponing can cost add to these costs considerably. Furthermore, due to their ancient histories and followers’ beliefs, religious pilgrimages, such as Hajj cannot be canceled, postponed, or relocated as the rituals of these pilgrimages must be completed on specific dates at specific sites. Therefore, it is important to find effective ways to organize these events with the necessary safety measures.

The volume of people in attendance in close proximity at these mass gatherings makes them good candidates for “super spreader” events. Additionally, these global events have the potential of spreading the disease to even more countries and even more rapidly. But the impact of canceling these events or allowing restricted entries, or hosting with no restrictions on the scale of the pandemic has not been measured yet. The purpose of this paper is to study the impact of Saudi Arabia’s decision to suspend the Umrah pilgrimage on the trajectory of COVID-19 at local and global levels. We propose homogeneous and stratified SEIR (Susceptible-Exposed-Infected-Removed) mathematical models to simulate the transmission of COVID-19 during the Umrah pilgrimage among the different groups of pilgrims and the residents in Mecca. For a more realistic simulation of the epidemic and to incorporate the stochastic nature of the disease transmission, we used the discrete-time stochastic version of the homogeneous and stratified SEIR models. Several epidemic scenarios were executed during the holy month of Ramadan on a daily basis. Safety and precautionary measures such as wearing masks, social distancing, sanitizing surfaces may directly reduce the infectious disease parameters, contact rates of individuals, and the transmission probability of the infection (Lindsley et al. 2020; Wang et al. 2020; World Health Organization 2009). We simulate the levels of the employed safety measures implicitly by varying these parameters. The model was calibrated using the expected numbers of international and domestic pilgrims arriving at Mecca and the daily reported number of COVID-19 cases at the same time interval. The objective of the study is to analyze various scenarios for better understand the importance of global mass gatherings suspension as a control measure during pandemics.

In the next section, we provide background and existing literature review on mass gatherings and infectious diseases, common epidemic mathematical models, and mass gatherings in Saudi Arabia. The COVID-19 situation and response in Saudi Arabia are also summarized in Sect. 2. The homogeneous and stratified models are described in Sect. 3, including the basic deterministic models and the discrete-time stochastic mathematical model. The obtained results for different epidemic simulations are shown in Sect. 4. Finally, we conclude the paper with a discussion on these results, limitations of the approach, and future work for this research.

## Background and literature review

### Mass gatherings and infectious diseases

Mass gathering is defined by WHO as “any occasion, either organized or spontaneous, that attracts sufficient numbers of people to strain the planning and response resources of the community, city or nation hosting the event.” (World Health Organization 2015). Mass gatherings (MGs), in general, can have serious public health consequences such as stampedes (Ahmed and Memish 2016), terrorist attacks (Steffen et al. 2012), and the spread of infectious diseases. The risk of disease transmission during global mass gatherings particularly, is mainly associated with three factors, social mixing, travel patterns, and the heterogeneity of the population of attendees and local residents of the host nation (Abubakar et al. 2012; Hopkins and Reicher 2020). The social mixing of a large number of people attending these events in confined settings for extended periods of time facilitate the disease spread among participants.

The travel and mobility patterns associated with MGs, whether these events are local, regional or global, can further contribute to the spread of infectious diseases across multiple locations at different scales. In times of pandemics, such as the influenza H1N1 pandemic in 2009 (Khan et al. 2009) and the ongoing COVID-19 pandemic (McCloskey et al. 2020), international travel caused by global MGs contributed to importing and exporting infections across multiple regions in the world in a short time (Alshammari et al. 2019). Participants and attendees in MGs are arriving from different locations, have different disease exposure histories, and diverse demographics. This mix of individuals with heterogeneous characteristics provides an ideal setting for the spread of various pathogens and diseases during such events.

Various public health measures, including vaccination requirements, screening at entry points, and event size restriction, can be implemented to contain the spread of diseases at MGs (World Health Organization 2015). For instance, the Hajj season of 2009 was during November in the middle of the influenza H1N1 pandemic, which caused many countries to prevent their citizens from performing Hajj that year (Ebrahim et al. 2009). However, sometimes these preventative measures and control strategies are not effective. For instance, screening at entry points is not capable of detecting asymptomatic participants upon their arrival at a mass gathering. The risk of infectious disease outbreaks at MGs presents an opportunity for researchers to develop epidemic models to simulate the spread of diseases in these settings. Epidemic models can be used in the planning process of MGs to estimate the potential risk of disease outbreaks during and after these events. Also, modeling infectious diseases provides a way to measure the sensitivity of the outbreak to various epidemic parameters, such as the initial number of cases, contact rate and patterns in the population, and the duration of the latent and infectious periods of the disease. Epidemic modeling can assist the decision making of epidemic response planning and the assessment of possible control measures.

### Mathematical modeling of epidemic diseases

**Fig. 1:**
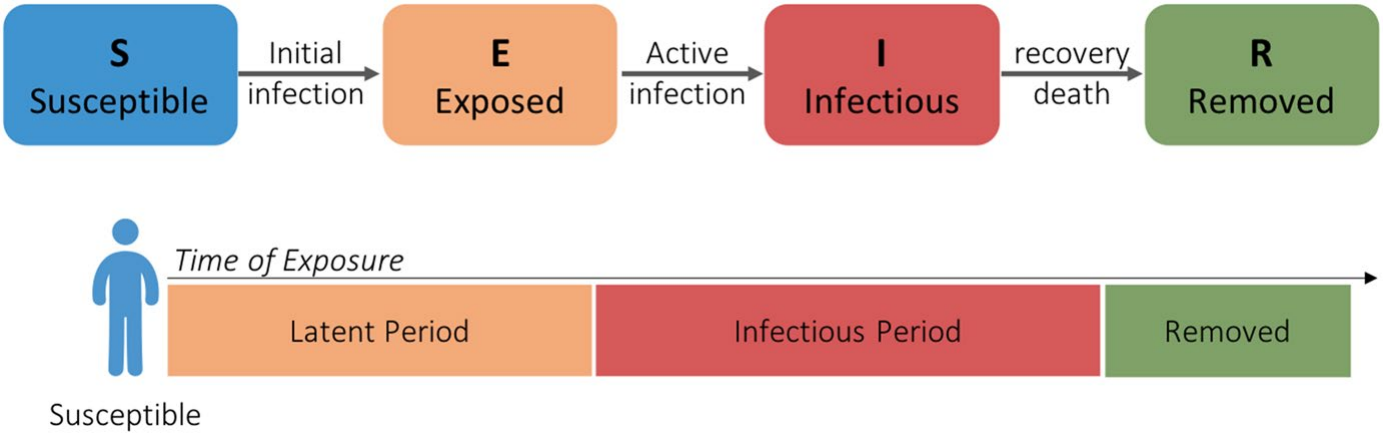
Compartments of the basic SEIR model, Susceptible (S), Exposed (E), Infectious (I) and Removed (R), corresponding to the progression of an infection in an individual over time

Epidemic models describe the spread of a disease in a population. Mathematical models and computer simulations have become important tools to analyze the spread and control of infectious diseases in different settings. In mathematical epidemic models, the underlying population is categorized into different compartments based on the infectious status of individuals. Each individual is then moved from one compartment to another based on the progression of the infection over time. In the basic SEIR model, the population is divided into four compartments: Susceptible (S), Exposed (E), Infectious (I) and Removed (R). These compartments correspond to the progression stages of an infection in an individual over time as illustrated in Fig. 1.

When a susceptible person (who is at risk of being infected) is exposed to an infectious agent or contacts an infected individual, he or she may be infected but is not infectious yet. A person will stay in this stage throughout the latent period of the disease. Then, the exposed individual will become infectious for the length of the infectious period. The infectious period starts with an individual being asymptomatic then the symptoms of the disease will appear. During this period, the infected person can transmit the disease to other susceptible individuals in the population. By the end of the infectious period, this individual will be removed, and no longer has a role in the progress of the disease transmission in the population. Depending on the disease, recovered individuals may gain permanent immunity to the disease. If no immunity is acquired, a recovered person will be susceptible and maybe be infected again. The removed stage can include recovered individuals and patients who die from the complication of the disease.

The SEIR model is based on the SIR (Susceptible-Infectious-Removed) model with an additional compartment for the Exposed population that is not infectious yet. Further extensions of the SEIR model were introduced to represent different status of the disease by including other compartments, such as (I_asym_) for asymptomatic infected individuals and (I_sym_) for infectious individuals who showed symptoms. Also, the removed compartment can include, in addition to recovered individuals, isolated infected individuals (either self-isolated or by public health officials). The SEIR model is the most commonly used model to study the progression of the COVID-19 outbreak. Variations of the basic SEIR model were presented in several studies to assess the impact of non-pharmaceutical measures (Goscé et al. 2020; Prem et al. 2020), simulate the spread of COVID-19 in specific settings (Rocklöv et al. 2020), or to predict the potential magnitude of the COVID-19 outbreak at local, regional, and global level (Hamzah et al. 2020).

### Global mass gatherings in Saudi Arabia

The major global mass gatherings hosted annually in Saudi Arabia are the two religious pilgrimages to the holy cities of Mecca and Madinah, Hajj and Umrah. These gatherings attract millions of Muslims from inside and outside Saudi Arabia. Hajj, the largest annual international gathering in the world, Muslims are expected to complete Hajj at least once in their life. The rituals of Hajj last for a few days during the last month of the Islamic calendar and are completed at multiple holy sites in Mecca including the great mosque (in Arabic called Al-Masjid Al-Haram). Umrah is considered to be a limited version of Hajj in terms of rituals and can be performed multiple times at any time of the year. The rituals of Umrah are limited to the great mosque in Mecca and can be performed in less than two hours. International and domestic pilgrims stay in Mecca during their visit to complete Umrah for a period that ranges between one day to three weeks. However, during the Hajj season international pilgrims can stay up to one month before the starting date of Hajj and one month after they complete Hajj. In both events, international pilgrims are allowed to visit the holy city of Madinah.

**Fig. 2:**
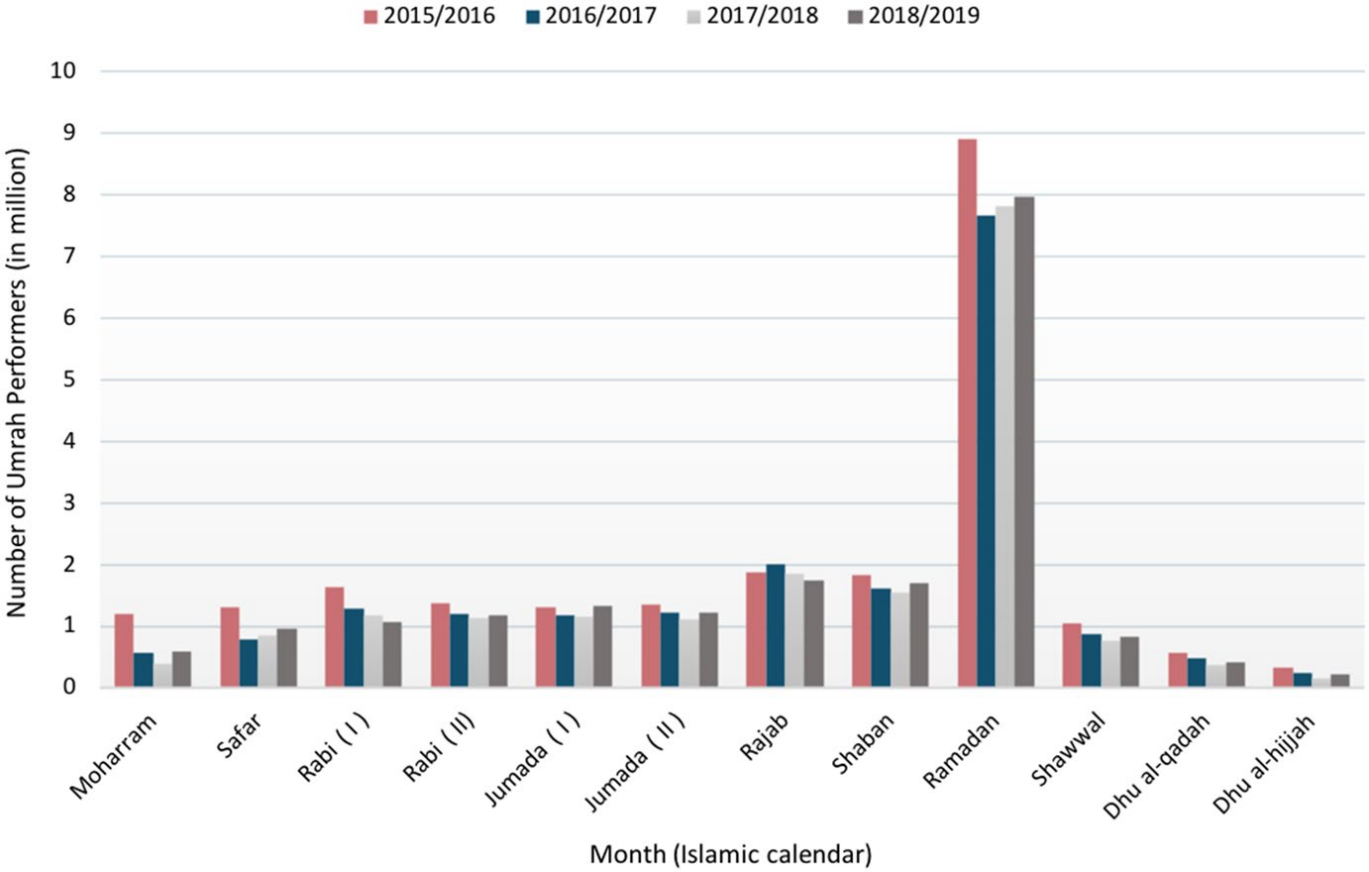
Number of international and domestic pilgrims per month for the past four Umrah seasons, between 2016 and 2019

In this study, we are focusing on the Umrah pilgrimage since we are studying the impact of the suspension of global mass gatherings on the spread of COVID-19 pandemic. The Umrah season starts approximately one month after Hajj and stays open until two to three weeks after the month of Ramadan (the 9th month of the Islamic year), for almost nine to ten months. Based on collected data of several Umrah seasons between 2011 and 2019 from different official sources, including the Saudi General Authority for Statistics (General Authority for Statistics 2020), and the Custodian of the Two Holy Mosques Institute for Hajj and Umrah Research (Custodian of the Two Holy Mosques Institute 2020), the peak period of the Umrah season is during the month of Ramadan with 40–43% of the total pilgrims performing Umrah during this month. Figure 2 shows the total number of domestic and international pilgrims per month for the past four Umrah seasons between 2016 and 2019.

We used the collected Umrah data to predict the expected number of international and domestic pilgrims in Ramadan of the 2020 Umrah season using the Exponential Triple Smoothing (ETS) algorithm, also called the Holt-Winters model. Exponential smoothing is a time series forecasting method commonly used for univariate data and short-term predictions (Kalekar et al. 2004). We based our prediction using only the past four Umrah seasons, between 2016 and 2019. Data before the year 2016 were excluded because the number of pilgrims in the previous Umrah seasons was restricted by the capacity of the great mosque of Mecca and due to the emergence of the Middle East Respiratory syndrome coronavirus (MERS-CoV) in 2012. The ETS forecasting algorithm estimated over six million (6,313,400) domestic pilgrims and over one million (1,305,180) international pilgrims in Ramadan 2020. The Umrah population mainly consists of domestic and international pilgrims. The terms pilgrims and Umrah performers are used interchangeably to describe the domestic and international visitors to Mecca to perform Umrah. In addition to pilgrims, Umrah population includes locals or residents of Mecca.

### COVID-19 situation in Saudi Arabia

Over the past years, the control measures applied by the Saudi government have contributed to the successful control and prevention of infectious diseases in the country, especially during the annual Hajj gathering. With a previous history of the MERS-CoV outbreak in 2012, Saudi Arabia’s response to potential epidemics has improved. In response to the COVID-19 pandemic, Saudi Arabia had taken unprecedented steps to slow the spread of the disease. As the disease continues to rapidly spread across the world, more intensive non-pharmaceutical measures were implemented such as border closure, cities lockdown, and full curfew nationwide (Algaissi et al. 2020). By enforcing international travel restrictions, suspension of Umrah, and restricting the 2020 Hajj season to a limited number of domestic pilgrims (Ebrahim et al. 2020), the Saudi government made courageous decisions to contain the global threat of COVID-19, and prevent importing the disease to other parts of the country, or causing further spread across the world.

**Fig. 3:**
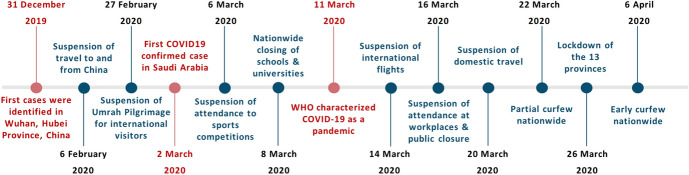
Timeline of the Saudi Arabia’s precautionary measures against COVID-19 illustrating major facts of the pandemic

With increasing COVID-19 cases in Saudi Arabia, additional measures were employed to prevent COVID-19 transmission within a city, a province, and between different regions in the country, such as district closures, citywide lock downs, and partial or full curfews. Along with widespread testing, these measures successfully identified regions and communities with active COVID-19 infections, thus, providing a geographical mapping and effective tracking of the COVID-19 spread over time and space. Figure 3 summarizes the major non-pharmaceutical measures implemented by Saudi Arabia to enforce social distancing and movement restriction in an attempt to contain this pandemic and minimize the number of new COVID-19 cases and potential failure of the health care systems in the country.

**Fig. 4:**
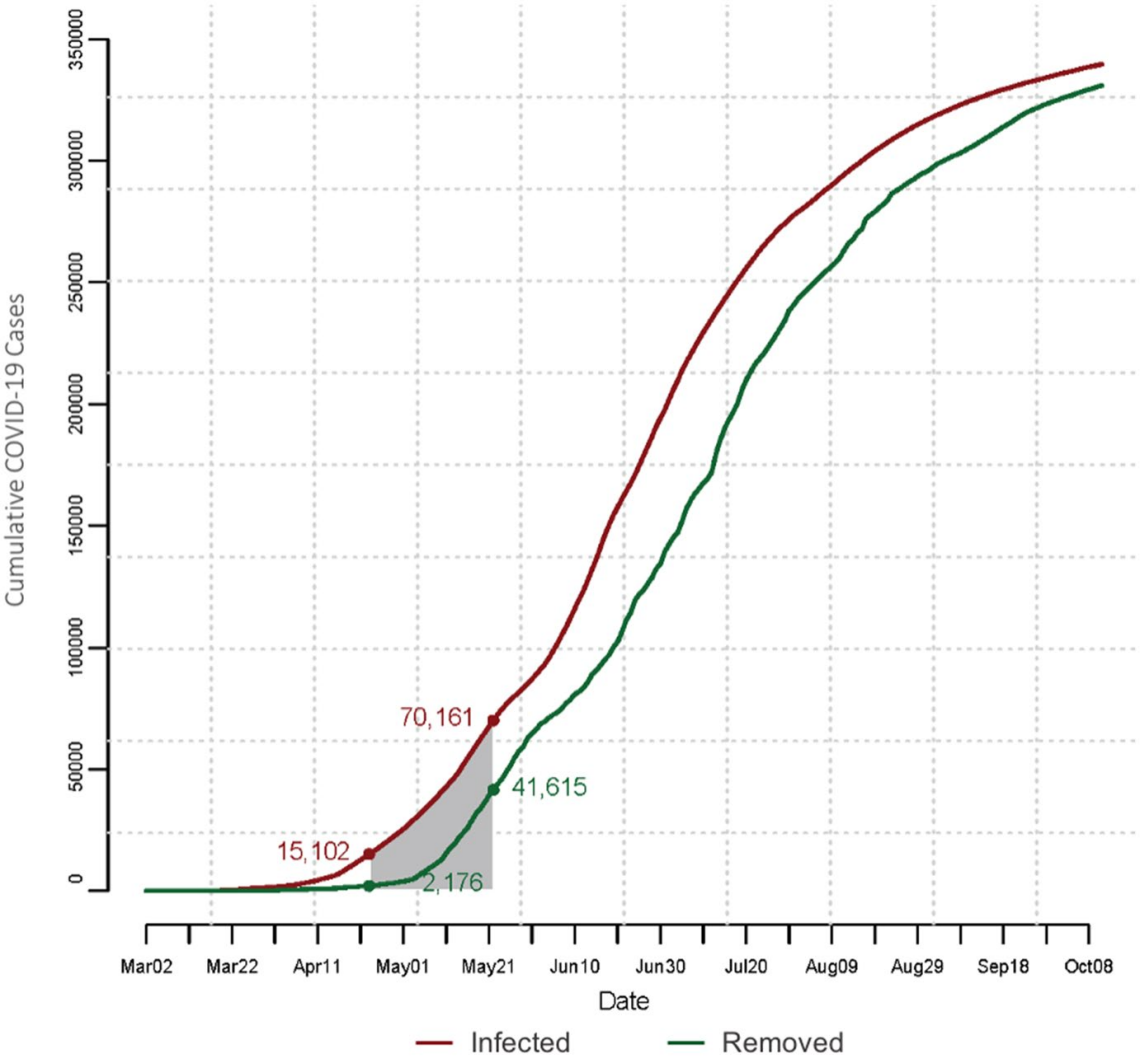
Daily updates of the cumulative number of confirmed and removed cases throughout the COVID-19 epidemic (From March 2, 2020 to October 11, 2020) in Saudi Arabia. Shaded area represents those numbers during the month of Ramadan

The cumulative number of COVID-19 cases, reported by the 11th of October in Saudi Arabia, reached 339,615 cases, including 8,709 active cases, 5,068 deaths attributed to COVID-19, and a total of 325,838 recoveries. The number of deaths in Mecca reached 781 cases, to be the third highest for a city in Saudi Arabia. Figure 4 shows the daily updates of the number of COVID-19 cases along with the number of removed cases from the start date of the ongoing COVID-19 epidemic in Saudi Arabia, March 2, 2020, until the 11th of October 2020. The shaded area in the diagram represents the number of cases during the month of Ramadan, indicating the cumulative number of confirmed and removed cases at the begging (April 23, 2020) and end of Ramadan (May 23, 2020).

## Methodology

### Description of the epidemic model

To evaluate the implications of the suspension of the Umrah global mass gathering in Mecca, we used the mathematical SEIR model. In this study, we implemented two versions of the SEIR model, assuming: (1) a homogeneous population, and (2) a stratified population. In both models, the population is assumed to be static, where dynamic factors like immigration, emigration, deaths and births are not taken into account.

#### Homogeneous SEIR model

Umrah population consists of sub-groups of pilgrims and residents of Mecca with different attributes and varying social mixing patterns. However, in the homogeneous SEIR model, we assumed individuals in the same compartment have the same characteristics with the assumption of a constant contact rate among all individuals in the population. The following system of differential equations described the transition of individuals between the four compartments (Susceptible, Exposed, Infectious, and Removed) over time in the homogeneous SEIR model.

$$\begin{aligned} \frac{dS}{dt} &= -\beta S(t) \frac{I(t)}{N} \\ \frac{dE}{dt} &= \beta S(t) \frac{I(t)}{N} - \sigma E(t)\\ \frac{dI}{dt}&= \sigma E(t) - \gamma I(t)\\ \frac{dR}{dt} &= \gamma I(t) \end{aligned}$$

Here, $S(t)+E(t)+I(t)+R(t)=N$, where *N* is the population size. The state variables at time *t* are: $\mathbf{S(t)}$, the number of susceptible individuals who do not have the disease but can be infected, $\mathbf{E(t)}$, the number of exposed individuals who were infected by the disease but are not infectious yet, $\mathbf{I(t)}$, the number of infectious individuals who become infectious, and are able to transmit the disease to other susceptible individuals, and $\mathbf{R(t)}$ which reflects the removed individuals. In the basic SEIR model, $\beta$ is the transmission rate that measures the likelihood of disease transmission from an infected individual to a susceptible individual. It includes the probabilities of contact and transmission, hence is defined as the product of the average contact rate (*C*) and the transmission probability (${\tilde{\beta }}$), such that, $\beta = {\tilde{\beta }}\times C$. The duration of the latent and infectious periods are denoted by $1/\sigma$ and $1/\gamma$, respectively.

#### Stratified SEIR model

To study the impact of the diversity among the population of pilgrims and residents in Mecca during Umrah, we stratified the population in Mecca during the month of Ramadan into three sub-populations: international pilgrims, domestic pilgrims, and local residents. The stratified SEIR model can be described as follows:

$$\begin{aligned} \frac{dS_{i}}{dt} &= -\sum _{j=1}^{k}{\beta _{ij} S_{i}(t) \frac{I_{j}(t)}{N_{j}}} \\ \frac{dE_{i}}{dt} &= \sum _{j=1}^{k}{\beta _{ij} S_{i}(t) \frac{I_{j}(t)}{N_{j}}} - \sigma E_{i}(t)\\ \frac{dI_{i}}{dt} &= \sigma E_{i}(t) - \gamma I_{i}(t)\\ \frac{dR_{i}}{dt}&= \gamma I_{i}(t) \end{aligned}$$

In the stratified SEIR epidemic model, we consider $\beta _{ij}$ as the transmission rate from population *i* to population *j*. The transmission rate between individuals from the same subgroup may be different from the transmission rate between individuals from different sub-populations on account of the varying inter and intra group contact rates. $\beta _{ij}$ in this model represents the adjusted transmission rate from group *i* to group *j*. The transmission rate is adjusted by a contact factor $C_{ij}$ that controls the number of contacts between the two sub-populations *i* and *j* such that, $\beta _{ij} = {\tilde{\beta }}\times C_{ij}$, where ${\tilde{\beta }}$ here is the probability of transmission as defined in the homogeneous model.

#### Stochastic SEIR model

For a more realistic simulation of the epidemic and to incorporate the stochastic nature of the disease transmission, we extended the deterministic SEIR models described above to a discrete-time stochastic version (Lekone and Finkenstädt 2006; He et al. 2020). In the stochastic version of the SEIR model, the transition rates between the different states in the model at time point *t* will be based on a binomial distribution. The new number of individuals in each state will be calculated based on the size of each state and the estimated transmission rate between that state and the next state at time *t*. Thus, at time $$[t, t+1)$$, the number of individuals that will move from a state to other will be sampled from the binomial distribution with a probability $$1-exp(-\lambda (t))$$, where $$\lambda (t)$$ represents the transition rate between the corresponding states that is deduced from the set of equations described in Sect. 3.1.2. The following set of equations define the simulation procedure of the stochastic SEIR model in a homogeneous mixing population at each time $$[t, t+1)$$.

$$\begin{aligned} S(t+1) &= S(t) - Q(t) \\ E(t+1) &= E(t) + Q(t) - M(t)\\ I(t+1) &= I(t) + M(t) - D(t)\\ R(t+1) &= R(t) + D(t) \end{aligned}$$

where *Q*(*t*) represents the number of newly exposed individuals affected by the infectious individuals at time *t*; *M*(*t*) represents the number of exposed individuals who become infectious at time *t*; and *D*(*t*) represents the number of infectious individuals who was removed at time *t*. Those numbers are simulated from the binomial distribution with probabilities as follows.

$$\begin{aligned} Q(t) &\sim Bin(S(t), 1-exp(-\beta \frac{I(t)}{N}))\\ M(t) &\sim Bin(E(t), 1-exp(-\sigma ))\\ D(t) & \sim Bin(I(t), 1-exp(-\gamma )) \end{aligned}$$

As shown in the following set of equations, we applied the same changes for the stratified version of the SEIR model:

$$\begin{aligned} S_{i}(t+1)& = S_{i}(t) - \sum _{j = 1}^{3}{Q_{ij}(t)} \\ E_{i}(t+1)& = E_{i}(t) + \sum _{j = 1}^{3}{Q_{ij}(t)} - M_{i}(t)\\ I_{i}(t+1)& = I_{i}(t) + M_{i}(t) - D_{i}(t)\\ R_{i}(t+1)& = R_{i}(t) + D_{i}(t) \end{aligned}$$

where $Q_{ij}(t)$ represents the number of newly exposed individuals in population *i* affected by infectious individuals in population *j* at time *t*; $M_{i}(t)$ represents the number of exposed individuals in population *i* who become infectious at time *t*; and $D_{i}(t)$ represents the number of infectious individuals in population *i* who was removed at time *t*. Those numbers are simulated from the binomial distribution, such that:

$$\begin{aligned} Q_{ij}(t) &\sim Bin(S_{i}(t), 1-exp(-\beta \frac{I_{j}(t)}{N_{j}}))\\ M_{i}(t) &\sim Bin(E_{i}(t), 1-exp(-\sigma ))\\ D_{i}(t) &\sim Bin(I_{i}(t), 1-exp(-\gamma )). \end{aligned}$$

### COVID-19 epidemic simulation

#### Parameters

The infection-related parameters for the SEIR epidemic model were inferred from published reports and studies about COVID-19 epidemiology (Liu et al. 2020; Wang et al. 2020; Wiersinga et al. 2020). We assumed the latent period to be 5 days $(\sigma = 0.2)$ and the infectious period to be 11.5 days $(\gamma = 0.087)$ long. The contact rate was estimated in the range of 7-10 based on previous studies of the pilgrims’ movements and contact patterns at the great mosque in Mecca (Dridi 2015), and on a mathematical contact model derived in (Hu et al. 2013) to estimate contact rates in high population density gatherings.

The reported values of the basic reproduction number, $R_0$, for COVID-19 ($R_0$ = 2.30-3.0 (Liu et al. 2020; Wang et al. 2020) can be used to infer the transmission probability of the disease from the relation between $R_0$ and the transmission rate where $R_{0} = {\beta }/{\gamma }$. $R_0$ is defined as the expected number of secondary cases or infections directly generated by the first infected individual in a completely susceptible population (Van den Driessche and Watmough 2008). $R_{0}$ is one of the epidemiological assessment metrics that can measure the severity of a disease epidemic in a population. A disease epidemic is said to take place only when the value of $R_0$ is greater than 1. Larger values of $R_0$ indicate more rapid spread of the disease. If the value of $R_{0}$ reaches below one, the disease outbreak is an endemic and is not considered a public health threat.

However, reported $R_0$ values were calculated based on COVID-19 contact tracing data obtained in specific settings. The severity of the COVID-19 pandemic and the growing numbers of new cases around the world, suggests high transmission rates for studying the spread of the disease in mass gatherings. Therefore, in the homogeneous SEIR epidemic model, we assumed the transmission rate $\beta$ to vary in a range of 0.2 to 0.7 by controlling the average contact rate (*C*) to 10 contacts per person, and using various values of the transmission probability (${\tilde{\beta }}$) ranging between 0.02 to 0.07. These values of $\beta$ resulted in a variation in the corresponding values of $R_0$ to range from 2.3 to 8.05.

In the stratified SEIR epidemic model, we incorporate the interactions between and within the sub-populations through the adjusted transmission rate $\beta _{ij}$. This rate is adjusted by the contact factor $C_{ij}$ that captures the average number of contacts between the two sub-populations *i* and *j*. Specifically, based on the average contact rate in the homogeneous model ($C = 10$), we differentiated the interactions among the different sub-populations with a higher contact rate (65%–75%) within a group and a lower number of contacts (25%–35%) directed to susceptible individuals in other groups. The transmission probability ${\tilde{\beta }}$ and corresponding $R_0$ values are identical to the homogeneous model.

Thus, as we considered here a heterogeneous contact rate $C_{ij}$ among individuals in the different sub-populations, we scaled the value of the transmission probability ${\tilde{\beta }}$ by the largest eigenvalue of the normalized contact matrix (Towers and Feng 2012), such that, $R_{0} = \frac{{\tilde{\beta }}}{\gamma } \times max(eigenvalue(M)),$ where *M* is the normalized contact matrix, such that, $M_{ij} = C_{ij}\times \frac{N_{i}}{N_{j}}$; $N_{i}$ and $N_{j}$ represent the size of the sub-populations *i* and *j* respectively.

#### Assumptions and initial conditions

We made a few assumptions to simplify the epidemic simulation during Umrah. These assumptions are described below:

1. Initial infection: Since the Saudi government imposes a visa restriction for international pilgrims to present a negative COVID-19 PCR test certificate we assume the initial infected individuals in the population are limited to domestic pilgrims and residents of Mecca.
2. Population size: As stated previously, international and domestic pilgrims varied in the length of their stay at Mecca. However, we assumed a static population with a fixed size throughout the duration of the simulation. Thus, from the start date until the end date of Ramadan, the number of Umrah performers is fixed without any new arrivals or departures from Mecca.
3. Simulation setting: We assumed that international and domestic pilgrims would spend most of their day in the great mosque in Mecca. Thus, the social mixing of the Umrah population is assumed to be higher in the great mosque and the surrounding areas where major hotels, shops, and restaurants are located. This assumption is justified by the fact that the rituals of the Umrah are completed inside the holy mosque, and pilgrims will be in the great mosque at least five times a day to perform the prayers. Furthermore, the movement and the accommodation settings of international and domestic pilgrims in Mecca during the month of Ramadan are not well documented, hence difficult to simulate without more data.
4. Disease states: We assumed that the removed compartment in the SEIR model includes recovered individuals and individuals who die from the complications of the disease. We also assumed that recovered individuals acquire permanent immunity to COVID-19, even though there are conflicting reports about recurrent COVID-19 infection after recovery. Individuals are assumed to stay in the exposed and infectious compartments throughout the latent and infectious periods respectively.

Given the parameters described in Sect. 3.2.1 and the assumptions presented above, the epidemic simulation in the conducted experiments was initiated from the first day of Ramadan (April 23, 2020) by seeding the residents of Mecca and the domestic pilgrims with infected individuals.

## Results

We executed several COVID-19 epidemic scenarios to study the impact of Umrah during the month of Ramadan by simulating the SEIR epidemic models for 30 days from the start day of Ramadan. In the first scenario, we tested the assumption of a homogeneous population of over 9 million individuals in Mecca without applying any restrictions on the movements of individuals, or on the number of the international or domestic pilgrims. Then, we examined the stratified version of the SEIR model assuming heterogeneous interactions among the different sub-populations of pilgrims (1,305,180 international and 6,313,401 domestic pilgrim) and the residents in Mecca (2,042,106 individuals). In both homogeneous and stratified stochastic SEIR models, 100 simulations were executed for each scenario to capture the effect of the stochasticity of the models. We report the mean number of individuals in each state across the 100 executions as the outcome for each scenario.

**Fig. 5:**
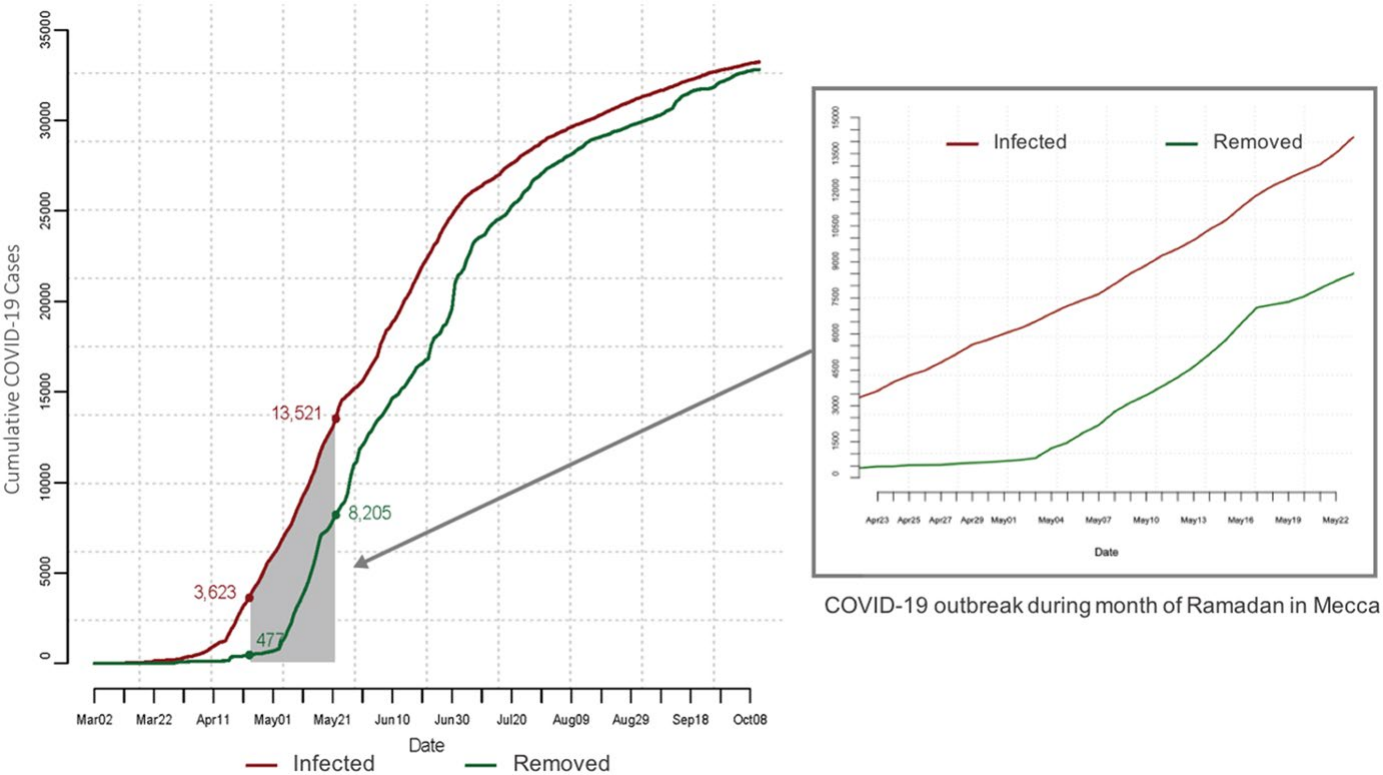
Daily updates of the cumulative number of confirmed and removed COVID-19 cases reported in the city of Mecca (From March 2, 2020 to October 11, 2020). Shaded area represents those numbers during the month of Ramadan

We used data of confirmed cases in Saudi Arabia prior to the start date of Ramadan (see Fig. 4) to initiate infections among domestic pilgrims, and the reported cases in Mecca (see Fig. 5) to initialize the number of infected from the residents of Mecca. The city of Mecca has observed 33,230 confirmed cases representing 9.78% of the total confirmed cases in Saudi Arabia (Fig. 5).

### Homogeneous SEIR model

**Fig. 6:**
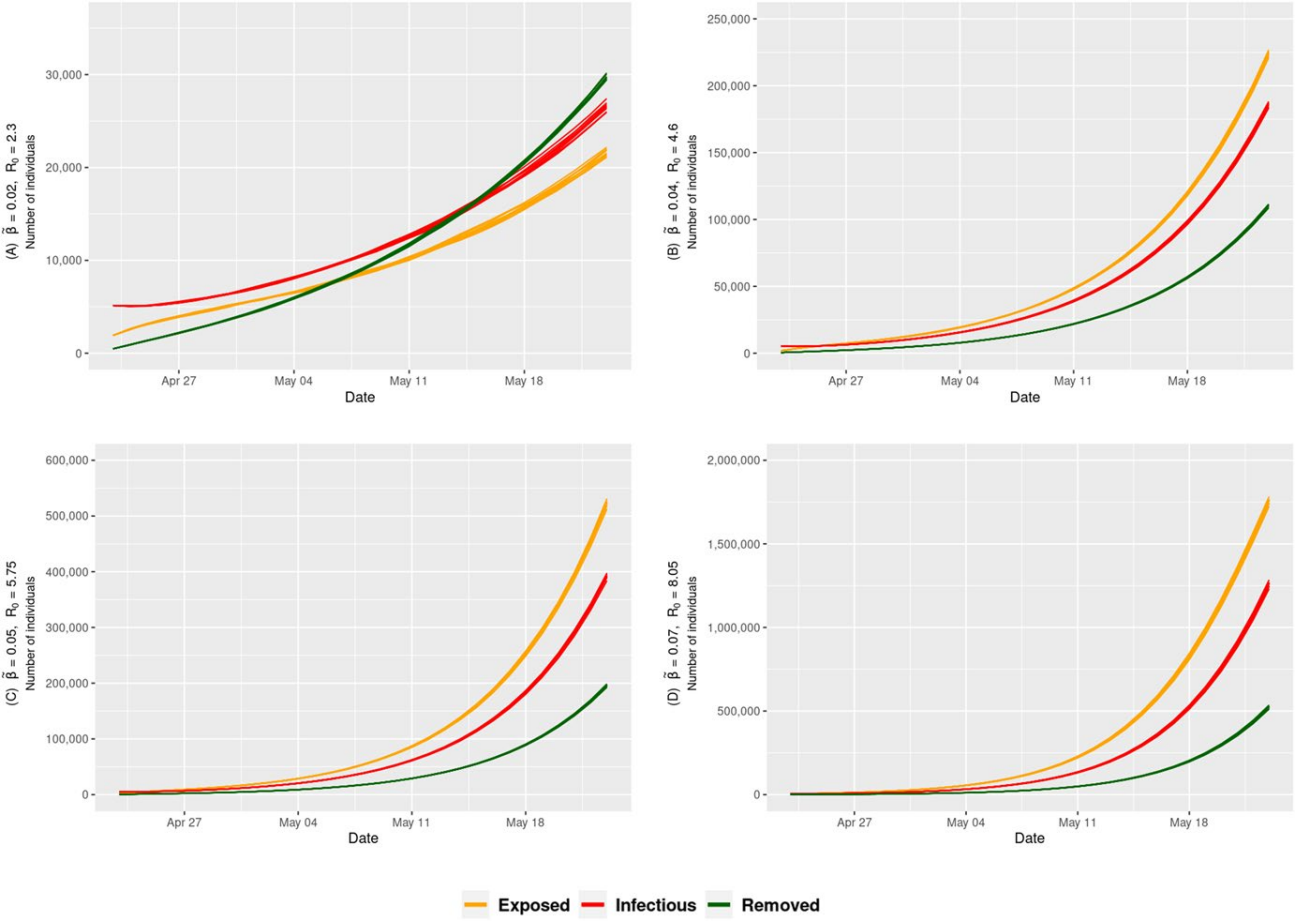
Estimated total number of infected individuals using the stochastic homogeneous SEIR model with different values of the transmission probability ${\tilde{\beta }}$ during the month of Ramadan in Umrah season without population size restrictions. The different values of ${\tilde{\beta }}$ simulate different levels of safety measures

Using the stochastic homogeneous SEIR model, we executed several simulations of the COVID-19 epidemic with different transmission rates. As shown in Fig. 6, our simulation of the spread of COVID-19 in Mecca using homogeneous mixing predicted an increasing cumulative number of infected cases. Table 1 shows the average number of predicted cases, including exposed, infectious and removed cases by the end of Ramadan. The table shows the effect of the transmission probability on the scale of the epidemic. A 3.5 times increase in transmission probability from 0.02 to 0.07 leads to a 50 times increase in the number of infected individuals from about 0.27% of the population to about 13% of the entire population. The number of individuals that can potentially spread the disease further (exposed and infectious) ranges between 47,000 to 3 million (Fig. 6) depending on the transmission probability. The results of the COVID-19 spread simulation using the homogeneous stochastic SEIR epidemic model with all the different values of ${\tilde{\beta }}$ is listed in Table 4 in Appendix.

**Table 1:** The average predicted number of cases by the end of Ramadan using homogeneous stochastic SEIR epidemic model at the best-case scenario ($R_{0} = 2.3$) and worst-case scenario ($R_{0} = 8.05$)

Scenario	Exposed	Infectious	Removed
Best-case	21,351.08	26,343.91	29,753.71
	(0.22%)	(0.27%)	(0.31%)
Worst-case	1,749, 774.05	1,253,657.50	521,335.50
	(18.11%)	(12.98%)	(5.40%)

### Stratified SEIR model

**Fig. 7:**
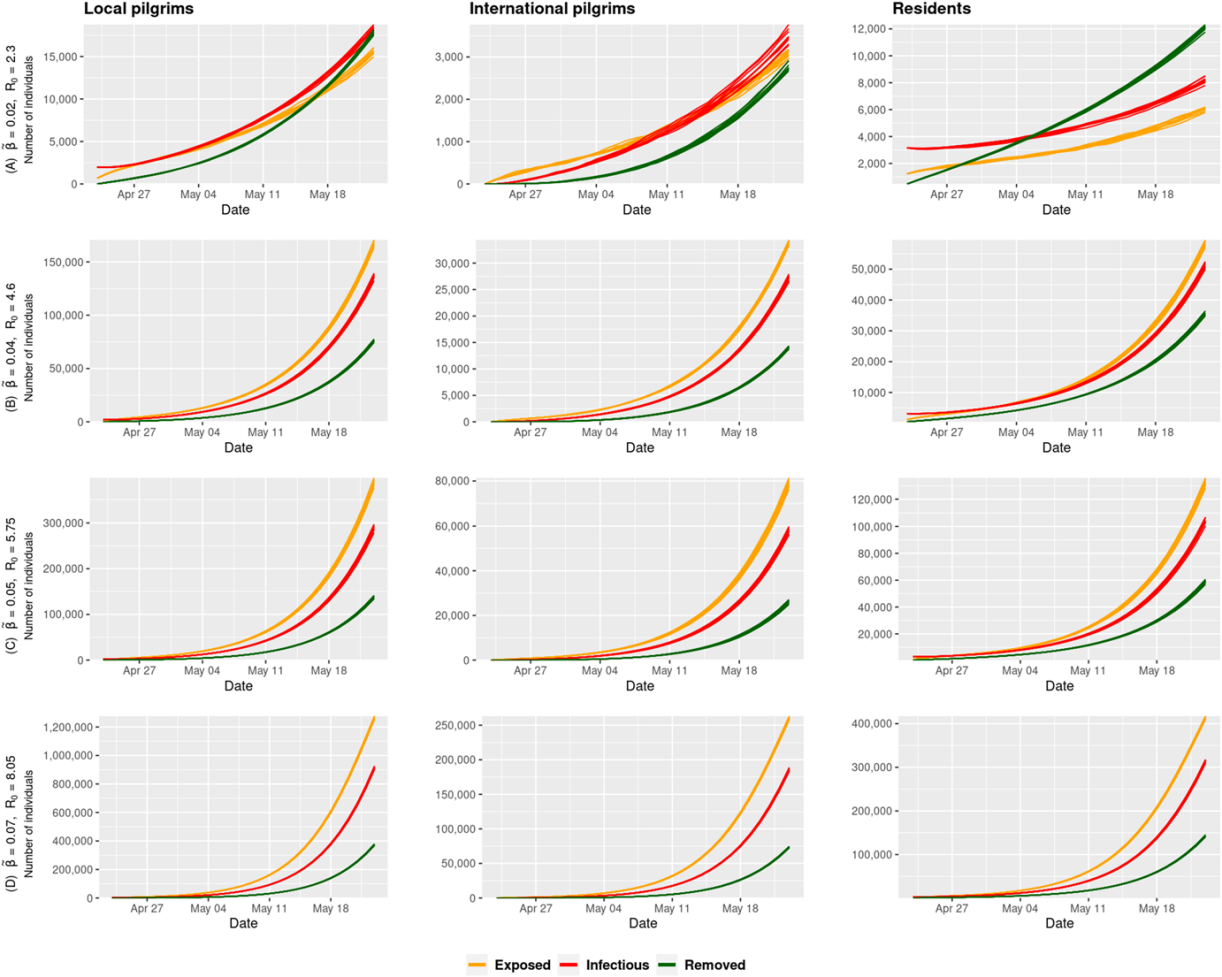
Predicted total number of infected individuals using the stratified SEIR model with different values of the transmission probability ${\tilde{\beta }}$ during the month of Ramadan in Umrah season

For more analysis of the impact of the heterogeneous interactions between the Umrah pilgrims and residents in Mecca, we simulated several COVID-19 epidemic scenarios using the stratified SEIR model applying the same sensitivity analysis of the transmission rate in the homogeneous SEIR model. However, rather than fixing the average contact rate, we considered a heterogeneous contact rate $C_{ij}$ among individuals in the different sub-populations. Figure 7 shows the epidemic curves for each sub-population under the different values of $\beta.$ As shown in the figure, the three sub-populations showed similar trends in their epidemic curves, which can be due to the similar distribution of contact rates between sub-populations. While we introduced the heterogeneity between the different sub-populations, we maintained homogeneous mixing within the same sub-population.

**Table 2:** The average predicted number of cases by the end of Ramadan using stratified stochastic SEIR epidemic model at the best-case scenario ($R_{0} = 2.3$) and worst-case scenario ($R_{0} = 8.05$)

Scenario	Population	Exposed	Infectious	Removed
Best-case	Mecca residents	5,999.84	8181.89	12,103.29
		(0.29%)	(0.40%)	(0.59%)
	Domestic pilgrims	15,572.86	18,412.46	17,821.41
		(0.25%)	(0.29%)	(0.28%)
	International pilgrims	3082.39	3470.51	2756.07
		(0.24%)	(0.27%)	(0.21%)
Worst-case	Mecca residents	416,359.75	315,478.94	143,875.91
		(20.39%)	(15.45%)	(7.05%)
	Domestic pilgrims	1,272,174.55	920,559.39	376,612.68
		(20.15%)	(14.58%)	(5.97%)
	International pilgrims	262,117.33	187,242.80	74,325.96
		(20.08%)	(14.35%)	(5.69%)

**Fig. 8:**
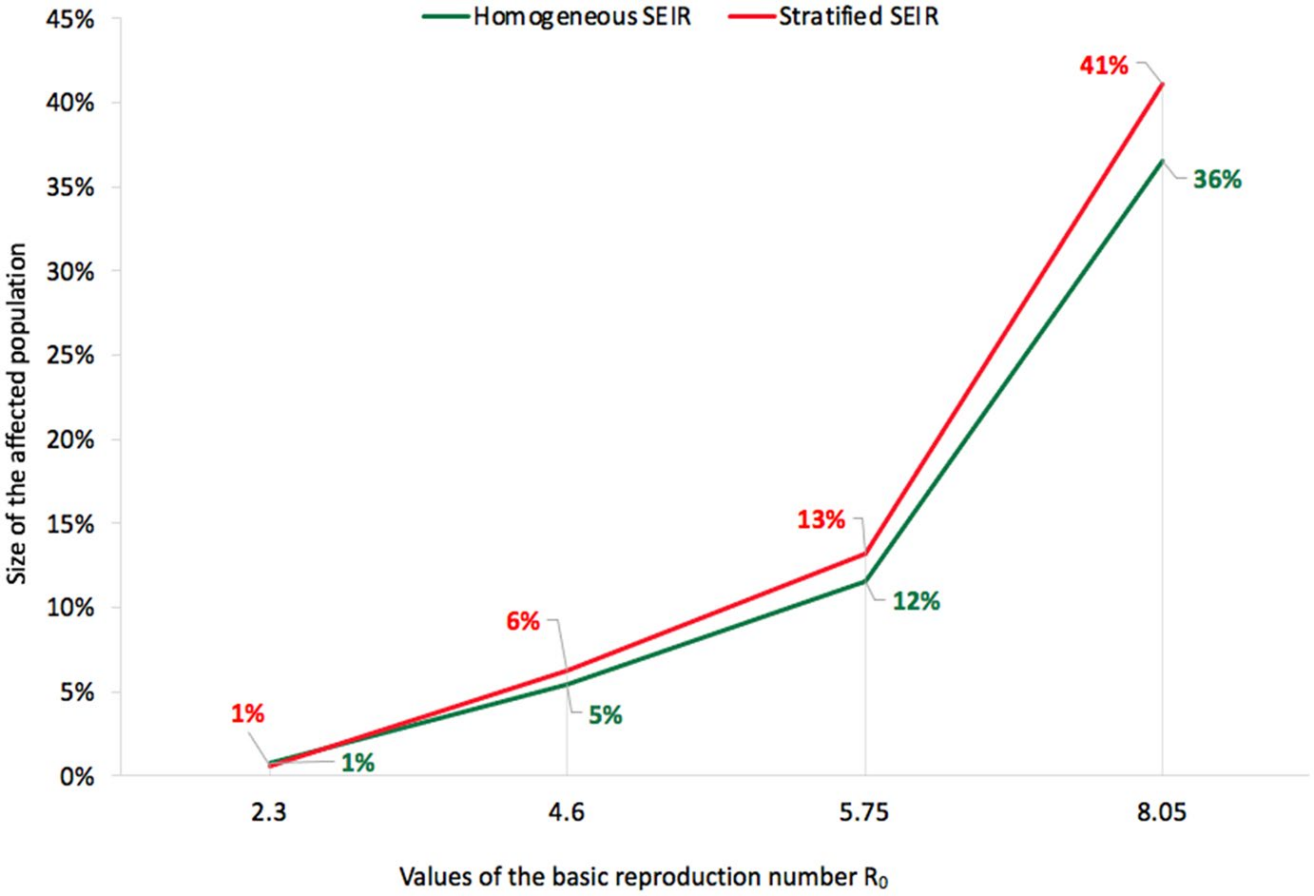
Comparison of the size of the affected population (exposed, infectious, and removed) relative to the total population between the homogeneous model and the stratified model for the different values of the transmission probability ${\tilde{\beta }}$ by the end of Ramadan in Umrah season

Results shown in Fig. 7 illustrate the effect of interactions among the different sub-populations of domestic pilgrims, international pilgrims, and residents on the disease spread among residents of Mecca. Table 2 shows the predicted total number of cases for each sub-population by the end of Ramadan. The simulation showed higher estimated cumulative cases compared to the observed COVID-19 cases in Mecca with the suspension of Umrah during the month of Ramadan. We see that the stratified model predicts a total number of affected population including exposed, infectious and recovered individuals would range between 0.90% to about 41% of the entire population in comparison to the 0.8% to 36% when using the homogeneous model. The results of the COVID-19 spread simulation using the stratified stochastic SEIR epidemic model with all the different values of ${\tilde{\beta }}$ are listed in Table 5 in Appendix. The stratification of sub populations does not have a major impact on the epidemic in the best case scenario of transmission probability, ${\tilde{\beta }} = 0.02$ as the reduction in effective contact rates nullifies the variation in the contact rates. However, the expected size of the epidemic is larger with stratification for higher values of ${\tilde{\beta }}$ as shown in Fig. 8.

While about 13,000 confirmed cases were reported in Mecca by the end of Ramadan, the simulation of the outbreak during Umrah estimated COVID-19 cases ranging between 6000 to 400,000. By the end of Ramadan, there could have been about 2 million domestic infected pilgrims and about 450,000 infected international pilgrims that could further spread the disease in their home cities and countries in the worst case scenario. However, the different scenarios in the simulation show that controlling the transmission probability can have a dramatic effect on controlling the size of the epidemic. If measures are taken to reduce the transmission probability by face coverings and social distancing, then the size of the epidemic can be reduced to less than 1% of the population.

These numbers reflect a significant impact of Umrah on further spread of the disease across multiple regions in the world. As of May 23, 2020 (end date of Ramadan), a total of 5,323,000 new COVID-19 cases were reported by WHO (World Health Organization 2020a) which may have been increased by about four million new cases related to pilgrims performing Umrah in Ramadan, where 13% (more than 450,000 cases) of them represents new cases of international pilgrims. Assuming that international flights are not suspended, the estimated numbers of new cases could have lead to a massive increase in the reported cases during the subsequent couple of weeks of Ramadan.

### Event size restriction

As stated previously, event size restriction is one of the main control measures that can be implemented to contain the spread of diseases at MGs (World Health Organization 2015). Thus, executing epidemic simulations using various numbers of domestic and international participants can provide insights into the effectiveness of limiting the number of attendees on the spread of the disease during this gathering. The stratified SEIR epidemic model was used to simulate the spread of COVID-19 between the population of pilgrims and the residents of Mecca when reducing the allowed number of international and domestic pilgrims by 25%, 50%, and 75%. We assumed that the reduction in the number of pilgrims is issued before the first day of Ramadan to maintain a static population throughout the simulation. Also, in these simulations, we did not enforce any restriction on the number of residents of Mecca by assuming any partial curfews and lockdowns. A corresponding reduction was applied to the initial cases in the domestic pilgrims’ population since the size of the residents is assumed to be unchanged.

The predicted numbers of cases at the end of the epidemic simulations showed that higher rates of size reduction in the pilgrims’ population could be an effective measure to control the disease spread during Umrah. The total number of exposed and infectious cases in pilgrims is reduced approximately by the same reduction rate in the pilgrims’ population compared with allowing all expected pilgrims to perform Umrah (0% size reduction). We try to measure the effect of the population size reduction along with the variation in the transmission probability (changes in $R_{0}$). Interventions such as use of masks, hand sanitizers and social distancing would ultimately help in reducing the transmission probability. Since the exact impact of these interventions is not known we use a range of values for $R_{0}$ between the lowest ($R_{0} = 2.3$) and the highest reported values ($R_{0} = 2.3$). We see that the reduction is attendees has a linear impact on the number of infected individuals. However, reducing the transmission probability in combination with the population size restriction can reduce the number of infected and exposed individuals to less than 25,000 individuals from about 3.5 million if no measures are taken, which points towards a possible controlled organization of the event. The number of infected and exposed international pilgrims is less than 2000 if 75% of the arriving population is restricted and the transmission probability is the lowest (best-case scenario). These individuals can be mandated to quarantine before their departures at this scale.

Table 3 lists the total numbers of the estimated COVID-19 affected population of pilgrims and residents of Mecca by the end of the 30 days of disease spread simulation when the value of $R_{0} = 8.05$. In this table, the impact of reducing the allowed numbers of pilgrims (by 25%, 50%, and 75%) is measured in terms of measuring the percentage decrease in the epidemic size compared when no changes are made to the initial numbers of local and international pilgrims. As shown in the table, the size of the epidemic is 2.4 times larger when no size restriction is made to the number of pilgrims than when reducing the number of pilgrims by 75%. The detailed results of the disease spread simulation with varying sizes of 100%, 75%, 50%, and 25% of the pilgrims population are listed in Appendix in Table 6 for the best-case scenario ($R_{0} = 2.3$) and the worst-case scenario ($R_{0} = 8.05$).

**Table 3:** The totals of predicted affected population, including pilgrims and residents of Mecca by the end of Ramadan using stratified stochastic SEIR epidemic model when reducing the number of allowed pilgrims by 25%, 50%, and 75% at the worst-case scenario ($R_{0} = 8.05$)

Size reduction	Exposed	Infectious	Removed	Total
0%	600,223.49	451,313.33	223,510.69	127,5047.51
25%	482,635.12	364,159.45	182,100.86	102,8895.43
	Epidemic size reduced by 20%			
50%	365,593.23	277,263.22	140,889.14	783,745.59
	Epidemic size reduced by 39%			
75%	248,538.36	190,438.49	99,685.44	538,662.29
	Epidemic size reduced by 58%			

## Discussion

Mathematical epidemic models can provide a preliminary assessment of the implications of mass gatherings in times of epidemics. Modeling the COVID-19 epidemic during Umrah, a global religious gathering in Saudi Arabia reveals important insights into the effectiveness of the suspension of mass gatherings during the spread of highly contagious diseases. The results of both homogeneous and stratified SEIR epidemic models suggested that the suspension of Umrah prevented an increase in the number of international and local COVID-19 cases compared to performing Umrah without isolation of infected pilgrims, or any restrictions in terms of the size of the pilgrims’ population. The obtained results of the COVID-19 spread simulation when applying size reduction to the local and international pilgrims showed the significant impact of restricting the allowed numbers of attendees on the overall size of the epidemic.

While the whole Umrah population was assumed to have similar features and same mixing patterns in the homogeneous SEIR model, the stratified SEIR model utilized more information stratifying the population into three sub-populations. Results of the predicted number of infected cases illustrated the effect of incorporating the interactions between these sub-populations in the stratified SEIR model. Tables 1 and 2 list the total number of predicted cases by the end of Ramadan in both homogeneous and stratified SEIR models. As shown in both scenarios, the best-case scenario (when $R_{0} = 2.3$) and the worst-case scenario (when $R_{0} = 8.05$), the total number of infected cases using the stratified SEIR model was greater than those using the homogeneous SEIR model. Incorporating more information to the model can enhance the granularity of the predictions.

Early decisions of suspending global mass gatherings, limiting the number of attendees, or restricting the participation to a specific population or group, are recommended during the spread of infectious diseases. Several factors, including the size and type of the gathering, and the demographics of the expected attendees, need to be taken into account in future modeling of the COVID-19 outbreak to evaluate the potential effectiveness of any health interventions on mass gatherings. Additionally, the timing of implementing these measures may also play a significant role in containing the spread of the disease.

## Limitations and directions for future research

The implementation of the mathematical epidemic model described in this research can be improved more by using a dynamic population rather than assuming a static population by including the arrival and departure of pilgrims throughout the simulation. The available Umrah data can be used to estimate the distribution of arriving and departing pilgrims to and from Mecca during the month of Ramadan. Executing the COVID-19 epidemic simulation with a dynamic population size can have a significant impact on the number of COVID-19 cases, as the mixing patterns and the contact rate may vary based on the population density. Additionally, the pilgrims can be stratified further based on country of origin or region with varying inter-strata contact rates. Due to limited data about the distribution of the international pilgrims at the country level and the domestic pilgrims at the regional level, the Umrah population was stratified into only three groups: international pilgrims, domestic pilgrims, and residents of Mecca. Also, epidermic mathematical models do not capture the spatial and temporal constraints of the pilgrim location and the dynamic behavior of the pilgrims.

Another area of improvement in the current implementation is in the distribution of the contact rate between the three groups. We can have more variations of the number of contacts, as in this study, we assumed same contact rates directed from one group to the two other groups. Also in this study, we limited the initial infection to domestic pilgrims and residents of Mecca. In the future, we can extend the initially infected individuals in the population to include asymptomatic international participants using the available COVID-19 data per country.

While the implemented mathematical models reveal important insights into the COVID-19 epidemic during Umrah, further extension of the methodology described in this study can be explored using stochastic agent-based models. Agent-based epidemic model can be implemented to simulate the COVID-19 pandemic in mass gatherings for effective evaluation of the impact of these settings at an individual level. In agent-based models, detailed attributes about the participating population can be included, such as country of origin, age-group, and health-related attributes. Agent-based models have been used in different studies to explore the effects of mass gatherings on the trajectory of the influenza pandemic (Shi et al. 2010) and the H1N1 pandemic (Alshammari et al. 2019).

Further simulations can be executed using dynamic sizes of the Umrah population, including domestic and international pilgrims and the residents of Mecca, to estimate the optimal population size to ensure social distancing and control the spread of the disease during the Umrah. Finally, implementing the restriction of the allowed numbers of participants or the suspension of Umrah can be further explored based on the time of employment of these interventions. These measures can be implemented at any time during a disease pandemic based on the severity of the outbreak outcomes. However, it is important to predict the optimal starting time to impose suspension or size restriction of a mass gathering. Thus, ensuring effective disease control while minimizing the social and economic consequences of these interventions.

## Appendix

**Table 4:** The average numbers of the COVID-19 spread simulation by the end of Ramadan using homogeneous stochastic SEIR epidemic model with different values of the transmission probability ${\tilde{\beta }}$

Scenario	Exposed	Infectious	Removed	Susceptible
$R_0 = 2.3$, ${\tilde{\beta }} = 0.02$	21,351.08	26,343.91	29,753.710	9,583,238.3
	(0.22%)	(0.27%)	(0.31%)	(99.20%)
$R_0 = 4.6$ , ${\tilde{\beta }} = 0.04$	225,734.1	187,046.49	111,023.55	9,136,882.86
	(2.34%)	(1.94%)	(1.15%)	(94.58%)
$R_0 = 5.75$ , ${\tilde{\beta }} = 0.05$	524,730.72	393,672.9	196,613.05	8,545,670.33
	(5.43%)	(4.07%)	(2.04%)	(88.46%)
$R_0 = 8.05$ , ${\tilde{\beta }} = 0.07$	1,749,774.05	1,253,657.5	521,335.5	6,135,919.95
	(18.11%)	(12.98%)	(5.40%)	(63.51%)

**Table 5:** The average numbers of the COVID-19 spread simulation by the end of Ramadan using stratified stochastic SEIR epidemic model with different values of the transmission probability ${\tilde{\beta }}$

Population	Exposed	Infectious	Removed	Susceptible
$$R_0 = 2.3,\;{\tilde{\beta }} = 0.02$$				
Mecca residents	5,999.84	8,181.89	12,103.29	2,015,820.98
Domestic pilgrims	15,572.86	18,412.46	17,821.41	6,261,594.27
International pilgrims	3,082.39	3,470.51	2,756.07	1,295,871.03
Total	24,655.09	30,064.86	32,680.77	9,573,286.28
	(0.26%)	(0.31%)	(0.34%)	(99.10%)
$$R_0 = 4.6, \;{\tilde{\beta }} = 0.04$$				
Mecca residents	58,630.47	51,314.28	35,591.34	1,896,569.91
Domestic pilgrims	167,304.76	136,434.48	76,011.28	5,933,650.48
International pilgrims	33,884.19	27,102.46	14,016.07	1,230,177.28
Total	259,819.42	214,851.22	125,618.69	9,060,397.67
	(2.69%)	(2.22%)	(1.30%)	(93.79%)
$$R_0 = 5.75,\;{\tilde{\beta }} = 0.05$$				
Mecca residents	132,558.16	104,506.7	58,992.21	1,746,048.93
Domestic pilgrims	388,473.35	288,849.23	138,150.14	5,497,928.28
International pilgrims	79,191.98	57,957.4	26,368.34	1,141,662.28
Total	600,223.49	451,313.33	223,510.69	8,385,639.49
	(6.21%)	(4.67%)	(2.31%)	(86.80%)
$$R_0 = 8.05,\;{\tilde{\beta }} = 0.07$$				
Mecca residents	416,359.75	315,478.94	143,875.91	1,166,391.4
Domestic pilgrims	1,272,174.55	920,559.39	376,612.68	3,744,054.38
International pilgrims	262,117.33	187,242.8	74,325.96	781,493.91
Total	1,950,651.63	1,423,281.13	594,814.55	5,691,939.69
	(20.19%)	(14.73%)	(6.16%)	(58.92%)

**Table 6:** The average predicted number of cases by the end of Ramadan using stratified stochastic SEIR epidemic model when reducing the number of allowed pilgrims by 25%, 50%, and 75% at the best-case scenario ($R_{0} = 2.3$) and worst-case scenario ($R_{0} = 8.05$)

Scenario	Population	Exposed	Infectious	Removed
0% reduction				
Best-case	Mecca residents	5,999.84	8,181.89	12,103.29
	Domestic pilgrims	15,572.86	1,8412.46	17,821.41
	International pilgrims	3,082.39	3,470.51	2,756.07
	Total	24,655.09	30,064.86	32,680.77
Worst-case	Mecca residents	416,359.75	315,478.94	143,875.91
	Domestic pilgrims	1,272,174.55	920,559.39	376,612.68
	International pilgrims	262,117.33	187,242.80	74,325.96
	Total	1,950,651.63	1,423,281.13	594,814.55
25% reduction				
Best-case	Mecca residents	6,016.83	8,197.97	12,117.61
	Domestic pilgrims	11,696.93	13,820.12	13,361.20
	International pilgrims	2,294.90	2,599.38	2,072.13
	Total	20,008.66	24,617.47	27,550.94
Worst-case	Mecca residents	416,326.68	315,454.04	143,894.02
	Domestic pilgrims	953,834.56	690,052.87	282,216.50
	International pilgrims	196,536.24	140,470.93	55,709.44
	Total	1,566,697.48	1,145,977.84	481,819.96
50% reduction				
Best-case	Mecca residents	6,009.70	8,210.04	12,114.22
	Domestic pilgrims	7,805.82	9,227.58	8,922.85
	International pilgrims	1,529.21	1,722.10	1,381.41
	Total	15,344.73	19,159.72	22,418.48
Worst-case	Mecca residents	416,417.80	315,714.36	143,854.03
	Domestic pilgrims	636,118.19	460,352.66	188,361.45
	International pilgrims	131,066.73	93,608.08	37,181.53
	Total	1,183,602.72	869,675.1	369,397.01
75% reduction				
Best-case	Mecca residents	5,994.08	8,170.35	12,087.39
	Domestic pilgrims	3,894.04	4,601.52	4,452.73
	International pilgrims	758.64	860.70	691.30
	Total	10,646.76	13,632.57	17,231.42
Worst-case	Mecca residents	416,431.18	315,619.13	143,932.21
	Domestic pilgrims	317,997.57	230,079.46	94,130.12
	International pilgrims	65,479.02	46,783.74	18,566.02
	Total	799,907.77	592,482.33	256,628.35
